# Personalized Medicine, Public Health and Patient-Centred Aspects in the Prevention of Stroke in Intracerebral Haemorrhage Survivors with Atrial Fibrillation (PRESTIGE-AF) Project

**DOI:** 10.1055/a-2576-7760

**Published:** 2025-04-30

**Authors:** Kirsten H. Harvey, Eleni Korompoki, Emily R. Harvey, Cornelia Fießler, Uwe Malzahn, Klemens Hügen, Sabine Ullmann, Carolin Schuhmann, Gabriele Putz Todd, Joan Montaner, Anna Penalba, Daisy Guaman-Pilco, Igor Sibon, Stephanie Debette, Timothy D'Aoust, Morgane Lachaize, Christian Enzinger, Stefan Ropele, Simon Fandler-Höfler, Viktoria Ruecker, Kirsten Haas, Peter B. Nielsen, Charles Wolfe, Yanzhong Wang, Hatem Wafa, Valeria Caso, Maria Giulia Mosconi, Gregory Y. H. Lip, Deirdre A. Lane, Walter E. Haefeli, Kathrin I. Foerster, Viktoria S. Wurmbach, Peter Ringleb, Peter U. Heuschmann, Roland Veltkamp, on behalf of PRESTIGE-AF investigators

**Affiliations:** 1 Department of Brain Sciences, Imperial College London, London, United Kingdom; 2 Department of Clinical Therapeutics, National and Kapodistrian University of Athens, Alexandra Hospital, Athens, Greece; 3 Clinical Trial Center Wüzburg, University Hospital Würzburg, Würzburg, Germany; 4 Department of Neurology, Institute de Biomedicine of Seville, IBiS/Hospital Universitario Virgen del Rocío/CSIC/University of Seville, Hospital Universitario Virgen Macarena, Seville, Spain; 5 Neurovascular Research Laboratory, Vall d'Hebron Institute of Research (VHIR), Hospital Vall d'Hebron, Barcelona, Spain; 6 University of Bordeaux, UMR-CNRS 5287, INCIA, Bordeaux, France; 7 Stroke Unit, Bordeaux University Hospital, Bordeaux, France; 8 UMR 1219 Bordeaux Population Health Center, University of Bordeaux, Bordeaux, France; 9 Department of Neurology, Institute for Neurodegenerative Diseases, Bordeaux University Hospital, Bordeaux, France; 10 Department of Neurology, Medical University of Graz, Graz, Austria; 11 Institute of Clinical Epidemiology and Biometry, University of Würzburg, Würzburg, Germany; 12 Department of Cardiology, Aalborg University Hospital, Aalborg, Denmark; 13 Department of Clinical Medicine, Faculty of Health, Aalborg University, Aalborg, Denmark; 14 School of Life Course and Population Sciences, King's College London, London, United Kingdom; 15 NIHR Applied Research Collaboration (ARC) South London, London, United Kingdom; 16 Stroke Unit - Internal, Vascular and Emergency Medicine, University of Perugia, Santa Maria della Misericordia Hospital Perugia, Perugia, Italy; 17 Liverpool Centre for Cardiovascular Science at University of Liverpool, Liverpool John Moores University and Liverpool Heart and Chest Hospital, Liverpool, United Kingdom; 18 Cardiovascular and Metabolic Medicine, Institute of Life Course and Medical Sciences, University of Liverpool, Liverpool, United Kingdom; 19 Internal Medicine IX - Department of Clinical Pharmacology and Pharmacoepidemiology, Cooperation Unit Clinical Pharmacy, Heidelberg University Hospital, Heidelberg, Germany; 20 Internal Medicine IX - Department of Clinical Pharmacology and Pharmacoepidemiology, Heidelberg University Hospital, Heidelberg, Germany; 21 Department of Neurology, Heidelberg University Hospital, Heidelberg, Germany; 22 Department of Neurology, Alfried-Krupp Krankenhaus Essen Germany; 23 Institute for Medical Data Science, University Hospital Würzburg, Germany

## Introduction

**Fig. 1:**
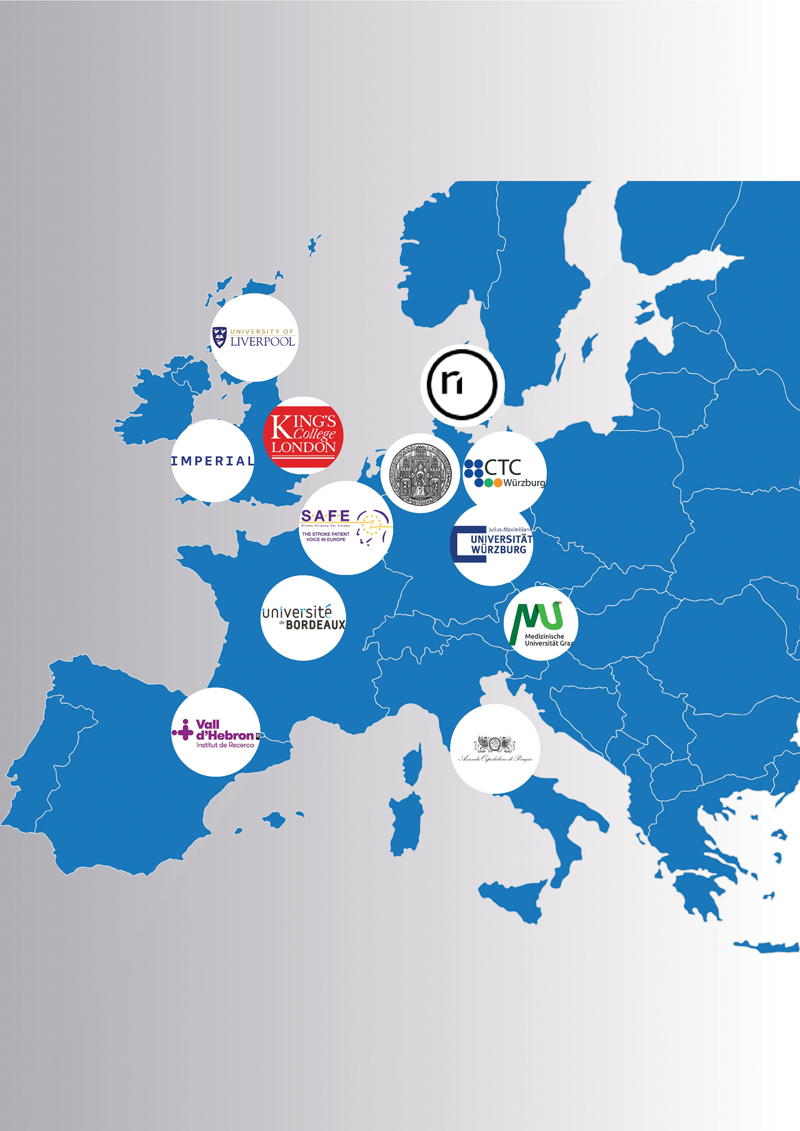
PRESTIGE-AF consortium institutions.

Although systematic reviews and meta-analysis of observational studies have suggested a benefit of oral anticoagulation (OAC) for ischaemic stroke prevention without a significant increase in recurrent ICH, experts agree that more evidence is required from randomized controlled trials (RCTs). 1 2 PRESTIGE-AF is a European Union Horizon 2020 funded project with a consortium of 12 institutions across 7 countries since 2019 ( Fig. 1 ). The aim is to explore optimal stroke prevention strategies for intracerebral haemorrhage (ICH) survivors with atrial fibrillation (AF), investigating the balance between the risk of ischaemic stroke against the risk of recurrent ICH.

**Fig. 2:**
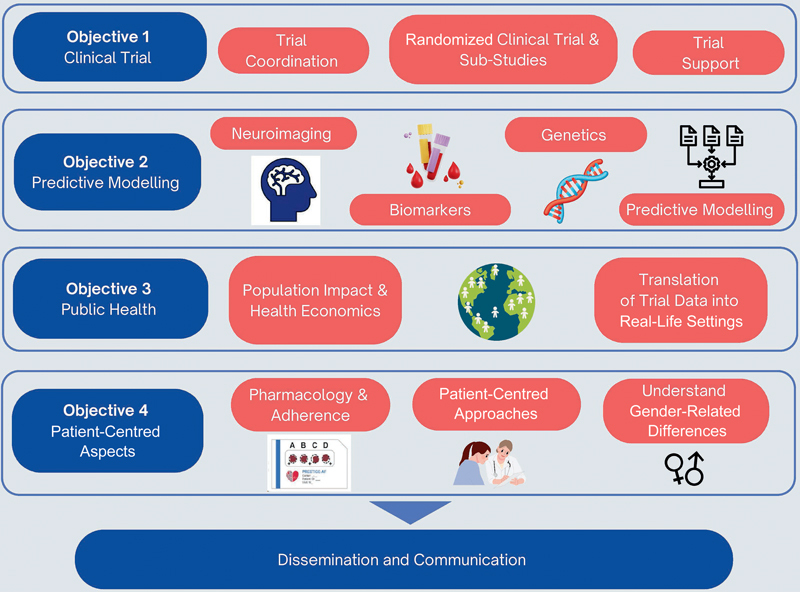
PRESTIGE-AF project overview.

The complexity of clinical management in ICH patients with AF is compounded by differences in clinical and patient characteristics, such as stroke severity, risk factors, and socio-demographics. Consequently, it is unlikely that RCTs alone will provide a solution for stroke prevention that will fit individual patients. Therefore, in addition to the main RCT, PRESTIGE-AF is examining how blood-based biomarkers, genetic factors, neuroimaging, and clinical features can be used to personalize stroke management. We are also exploring patient-centred aspects which can influence medication use and clinical trial participation. The project overview is shown in Fig. 2 . Clinical trial results can have a broad impact on health policy. This will be addressed within the project by modelling the health economic impact of our results and exploring if our findings are generalizable to a European population.

## Randomized Clinical Trial

The PRESTIGE-AF randomized clinical trial used a prospective, randomized, open, blinded end-point assessment (PROBE) design to determine the optimal antithrombotic management in patients with ICH and AF. Participants in the intervention arm received a direct oral anticoagulant (DOAC) compared with a control group who received either no therapy or an antiplatelet (at the investigator's discretion). Randomization occurred in a 1:1 ratio, stratified according to sex and ICH location. Between May 2019 and November 2023, a total of 319 participants were enrolled by 63 recruiting sites across 6 European countries and followed-up for a maximum of 36 months. The co-primary endpoints of ischaemic stroke and recurrent ICH will be tested hierarchically.

## Personalized Prevention Strategies

PRESTIGE-AF is also exploring personalized stroke prevention strategies using blood-based biomarkers, genetics, pharmacological, centrally analyzed neuroimaging, and a clinical risk prediction model.

Immunoassay techniques are being used to assess blood-based biomarkers (including NT-proBNP, cTn-hs, GDF-15) in a subgroup of the clinical trial participants who provided informed consent to evaluate ischaemic and ICH risks. A smaller proteomic study will also explore new biomarkers for ischaemic stroke and ICH, with promising candidates validated in the full cohort. PRESTIGE-AF has created a stroke patient blood biobank, both for discovery of new biomarkers and for testing of known candidates. We aim to develop a point-of-care device to apply these biomarkers in clinical settings, providing a tool to guide anticoagulation therapy and improve personalized treatment.

Using a subgroup of participants who underwent genetic testing, we are investigating whether AF or stroke-associated genetic variants, combined in polygenic risk scores (PRS), are related with risk of ischaemic stroke or ICH recurrence in our cohort. We are additionally investigating the association of MRI markers of cerebral small vessel disease (cSVD) PRS with risk of ICH recurrence or risk of ischaemic stroke.

It is well documented that blood concentrations of DOAC vary across patients; however, there is little evidence on the effect of this on patient outcomes, and the implication for dose adjustments. 3 A substudy in the intervention arm of the trial involves extensive characterization of the interindividual and interpatient variability of DOAC pharmacokinetics using DOAC blood concentrations derived from dried blood spots. The data from PRESTIGE-AF will explore if these techniques have a possible future clinical use.

Magnetic resonance imaging (MRI) is critical to determine the etiology of ICH, most frequently cSVDs such as arteriolosclerosis and cerebral amyloid angiopathy. 4 The presence and severity of these are key predictors of future risk of recurrent ICH and ischaemic stroke, 5 and the central assessment of MRIs in the clinical trial have allowed for characterization of individual risk profiles. A subgroup of clinical trial participants underwent MRI follow-up after 1 year to explore how neuroimaging factors may be used to support individual therapeutic decisions. The presence and severity of these are key predictors of both future risk of recurrent ICH and ischaemic stroke. 5 Central assessment of MRI will allow characterization of individual risk profiles. Additionally, uncovering dynamics of underlying cerebral small vessel disease may also hold promise and support individual therapeutic decisions. For this purpose, a subgroup of patients has undergone standardized MRI follow-up 1 year after baseline to add dynamic subclinical components of information.

For patients with AF established clinical risk scores such as the CHA _2_ DS _2_ -VASc score for thromboembolism, and HAS-BLED score for bleeding, are widely used in guidelines globally 6 7 8 but there are limitations in their use after ICH. 9 10 For instance, the HAS-BLED score does not include factors such as cerebral microbleeds. 11 In PRESTIGE-AF, a clinical risk prediction model is being developed to estimate the risk of poor outcome at 6 months and the risk of ischaemic stroke, ICH, and mortality within 12 months. A basic risk model will be determined using demographics, medical history, and clinical information from the index ICH and will be externally validated. If the sample size is sufficient, information from neuroimaging, genetics, and biological data will be incorporated to investigate improvement of risk prediction of the basic risk model.

## Public Health

PRESTIGE-AF will also address the European and global population impact of the trial findings. The Global Burden of Disease (GBD) (1990–2010) showed a 47% increase in absolute number of people suffering from ICH, the number of related deaths (20%), and disability adjusted life years lost (14%). 12 Systematic reviews have found no change in case fatality in population-based studies over several decades from 1 month (40%) to 5 years (71%) after ICH, although improvements have been noted in some individual populations. 13 14 Multivariate survival analysis has estimated a hazard ratio of 1.44 for AF and 1.21 for ICH and 1.45 for all-cause mortality in first ever stroke patients. 15 16

Currently, it is unclear whether existing recommendations are applicable to this population, and whether the predictive accuracy of established scoring systems holds in this context. Our analysis of GBD data predicts that deaths from ICH will increase by 8.9% across Europe by 2050—with some countries expected to have an increases of up to 74.4%. 16 Our analysis of Danish registry data has revealed a high risk of cerebrovascular ischaemic events and a markedly elevated risk of all-cause mortality within 1 year of the initial ICH. 17

Outputs from the PRESTIGE-AF clinical trial are essential for determining the most effective stroke prevention strategy for ICH survivors with AF. To advance this goal, we will use registry data to externally validate a prediction model based on the trial data, potentially guiding a new treatment approach for this vulnerable population. 18 19 20 21

Stroke has a major economic impact on communities, families, and the health system. PRESTIGE-AF will model the health economic impact of the trial findings using process of care data, quality of life (QoL), survival and activity outcome measures, along with specific outcome measures selected for this analysis. The results will contribute to decisions surrounding preventive strategies that are medically effective and more cost efficient.

## Patient-centred Aspects

An important component of preventive medicine is understanding factors that could influence uptake and adherence to medication. We explored attitudes to medication using patient questionnaires in the clinical trial and qualitative interviews with patients and physicians. Additionally, we are investigating cognitive function, anxiety, depression, and QoL, across the follow-up visits in the clinical trial.

Gender is an important aspect that is often forgotten in clinical trials. The underrepresentation of women in stroke prevention research can lead to results not being as generalizable to women. 22 23 Sex differences in stroke associated with common risk factors such as atrial fibrillation have gained attention recently, 24 with proposal of the non-sex CHA _2_ DS _2_ -VASc (i.e., CHA _2_ DS _2_ 2-VA) score.

We have surveyed the factors contributing to this and will use the results to identify ways to increase enrolment of women into future trials, such as educational campaigns and support networks which address gender-specific challenges in trial participation.

## Discussion

An emerging theme identified through qualitative interviews with physicians was ‘managing uncertainty’. 25 Antithrombotic treatment decisions were perceived as challenging due to clinical equipoise based on the current lack of robust clinical trial evidence, with decisions guided by patients' ability and willingness to engage with OAC; functional status; and associated co-morbidities. Interviews with patients showed that they prioritize their QoL when deciding about their stroke preventative treatment and their decisions were impacted by their individual beliefs about health, trust in their healthcare team, and input from their family. A discussion aid was created to facilitate shared decision-making between healthcare professionals and patients, to promote patients' understanding of their future stroke and bleeding risk. 26

Data from two pilot studies, APACHE-AF and SoSTART, suggest that anticoagulation protects against major ischaemic events but that this may be offset with an increased risk of haemorrhagic complications. 27 28 More robust clinical evidence demonstrating the efficacy and safety of OAC in patients with ICH and AF is required. The PRESTIGE-AF clinical trial is expected to generate important insights into the best antithrombotic management of ICH survivors with AF. The trial data will be shared for individual patient-data prespecified meta-analysis to broaden the evidence on this topic for informing clinical guidelines. 14 Beyond quantitative balancing of these opposing risks, tools to personalize decision-making in this complex situation are needed, with individual risk profiles having the potential to strongly influence those decisions using data from this trial.
